# Access to adolescent-responsive oral, mental, sexual, and reproductive healthcare services in Africa through dental clinics

**DOI:** 10.3389/froh.2025.1545988

**Published:** 2025-04-30

**Authors:** Nadia Adjoa Sam-Agudu, Chinye Osa-Afiana, Maha El Tantawi, Moréniké Oluwátóyìn Foláyan

**Affiliations:** ^1^ International Research Center of Excellence, Institute of Human Virology Nigeria, Abuja, Nigeria; ^2^ Department of Paediatrics and Child Health, University of Cape Coast School of Medical Sciences, Cape Coast, Ghana; ^3^ Global Pediatrics Program and Division of Infectious Diseases, University of Minnesota Medical School, Minneapolis, MN, United States; ^4^ Faculty of Dentistry, Alexandria University, Alexandria, Egypt; ^5^ Oral Health Initiative, Center for Reproduction and Population Health Studies, Nigerian Institute of Medical Research, Yaba, Lagos, Nigeria; ^6^ Africa Oral Health Network, Alexandria University, Alexandria, Egypt; ^7^ Department of Child Dental Health, Obafemi Awolowo University, Ile-Ife, Nigeria

**Keywords:** adolescents, delivery of healthcare, health service integration, dental clinics, HIV, Africa

## Abstract

In many healthcare systems, oral healthcare is provided separately from other clinical services. For 10–19-year-old adolescents in particular, this separation of care perpetuates the underutilization of oral health services and the neglect of oral health. Available evidence indicates that there are interconnections between oral, mental, sexual, and reproductive health (OMSRH) in adolescents. For African countries, there are opportunities to draw on lessons learned from HIV-centered models of integrated care to develop and evaluate dental clinic-centered models for integrating adolescent OMSRH services. This article makes a case for evidence-based adolescent OMSRH service integration in African countries. Integration is expected to align with the principles of sustainable development goals, universal healthcare, and the World Health Organization's calls for adolescent-responsive health services. We present a conceptual framework and propose an implementation science-guided blueprint for the integration of adolescent OMSRH care. The focus on dental clinics for integration can potentially increase access to, and use of oral healthcare while addressing adolescents’ mental, sexual and reproductive health needs. OMSRH integration for adolescents in African settings will require intensive engagement of adolescents and other crucial stakeholders. Further exploratory and implementation research is also needed to design and evaluate OMSRH integration models to establish best practices for long-term impact on adolescent health outcomes.

## Introduction

In many healthcare systems, oral healthcare is provided separately from other clinical services (1). To achieve the goals of universal healthcare, and to increase efficiency in the use of public health resources, health programs in different countries are increasingly integrating oral health services (2). The World Health Organization (WHO) Global Oral Health Action Plan also calls for the integration of oral health within the non-communicable disease (NCDs) agenda (3). In low and middle-income countries (LMICs) with high HIV burden, health service integration has been propelled by the need to improve the quality of HIV services. The evolution of care and support services for HIV has seen the integration of mental health, sexual and reproductive health (SRH), and maternal and child health with HIV services (4–6). However, there are few precedents for oral health services integration in high HIV-burden LMICs (7) despite the growing recognition of the benefits of integrating critically needed, poorly accessed services for different populations (8).

Adolescents are one of the populations that need focused attention for their health care needs. Care is needed for their developing mental, physical, and sexual health as they transit to adulthood. In this respect, the WHO called for investments beyond adolescent-friendly services to adolescent-responsive services, where (1) the developing capacity and autonomy of adolescents are acknowledged and addressed; (2) there are anticipatory models of care that can effectively screen for and manage health risks within adolescents’ daily context; and where (3) there are adolescent-competent providers, adolescent-protective financial policies and adolescent-responsive health information systems (9). Adolescents have also called for clinics and health systems to provide adolescent-focused and adolescent-responsive care (10–14).

The delivery of integrated, adolescent-focused oral, mental, and sexual, and reproductive health (OMSRH) services within dental clinics is a relatively new concept. However, this concept aligns with the WHO's eight priority areas for adolescent health in LMICs for 2030: (1) communicable diseases; (2) injuries and violence; (3) mental health; (4) non-communicable diseases; (5) nutrition; (6) physical activity; (7) substance use, and (8) policy, health and social systems affecting adolescent health (15). SRH research priorities are addressed separately (16). Although oral health was not explicitly mentioned, oral diseases are NCDs, and share common risk factors with other NCDs (17). Oral health is, however, poorly recognized, and poorly addressed in research, policy, or practice. In addition, oral health research is often not policy-driven, and oral health policy is often not research-based (18). Adolescent-relevant policies and practices are also often devoid of oral health content. Integrating oral health care with general health services for adolescents can help bridge this gap by ensuring that oral health is recognized as a critical component of overall well-being. By embedding oral health within broader adolescent-focused health services, including mental, sexual, and reproductive health, healthcare systems can address shared risk factors, improve accessibility, and promote holistic adolescent care. Strengthening policy frameworks to incorporate oral health into adolescent health agendas will be essential for achieving sustainable improvements in both research and practice for te population.

**Figure 1:**
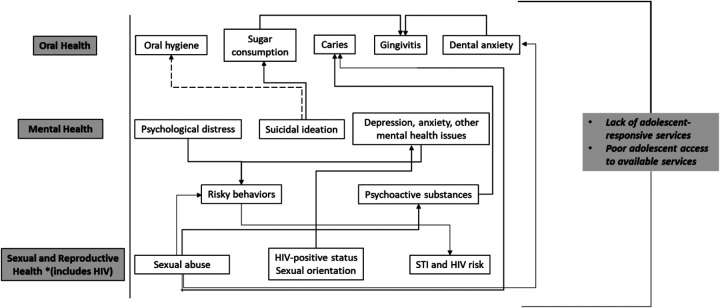
Conceptual framework depicting interconnections between oral, mental, sexual and reproductive health among adolescents. Solid lines: relationship involves increasing or facilitating the outcome in the direction of the arrow. Broken line: relationship involves decreasing or limiting the outcome in the direction of the arrow.

Our team and others have published reports suggesting the interconnectedness of the various needs and risk factors for OMSRH in adolescents, which present an opportunity for integrated care in these areas (19–24). Figure 1 presents a conceptual framework explaining the interconnection between OMSRH among adolescents, based on evidence generated by our team in Nigeria, and by other researchers. The conceptual framework suggests that poor sexual health among adolescents is a risk factor for poor oral, mental, and reproductive health (24, 25). Similarly, mental ill-health is a risk factor for sub-optimal oral, sexual, and reproductive health (24). Finally, poor reproductive health is associated with sub-optimal oral and mental health (26), and poor oral health status is linked to poor mental and sexual health (27).

Interconnections between the OMSRH of adolescents—especially in African countries—may help explain the high prevalence and burden of these three health problems in this population. In 2019, nearly one third (31.0%) of adolescents across Africa had untreated caries in permanent teeth (28). In the same period, the prevalence of untreated decay in permanent teeth among 10–19-year-old adolescents in the WHO African Region (AFRO) was 29.8%, and in the Middle East and North African (MENA) region was 39.9%. In addition, the prevalence of periodontal disease among adolescents in Africa was 1.6%; 1.7% in AFRO and 0.5% in the MENA region (28). The establishment of a variety of health insurance initiatives have not significantly reduced the prevalence of oral diseases in this population (26) indicating that upstream policy changes may be needed (29). In addition, adolescents' oral health behavior is significantly influenced by parental and peer influence in ways that are still not well understood (30).

Adolescent SRH is also driven by factors similar to those that drive oral health. The prevalence of sexually transmitted infections (STIs)—including HIV—among 10–19-year-old adolescents across Africa in 2019 was 7.0%: 7.6% in AFRO, and 3.6% in the MENA region (28). In these regions, adolescents face issues such as early pregnancy and parenthood, difficulties accessing contraception and safe abortion, and high rates of HIV and STIs (31). Economic, political, and sociocultural factors peculiar to the African contexts have limited the delivery of supportive, non-judgmental, youth appropriate SRH services in African countries (31).

These factors have also limited adolescents' access to quality mental health care, contributing to chronically undertreated mental health disorders that continue into adulthood, sometimes manifesting as highly disruptive and debilitating conditions (32). In 2019, it was estimated that 12.8% of 10–19-year-old adolescents in Africa had mental health disorders: 11.9% of adolescents in AFRO and 18.5% in MENA (28).

In this article, we explore and propose an approach for integrated OMSRH care for adolescents in Africa. We propose a paradigm shift in the delivery of integrated health services delivery wherein the dental clinic becomes the service delivery point for integrated care of adolescents. In doing so, we expect to increase access to and uptake of oral health services, a chronically neglected area of care, while also improving adolescents' access to comprehensive SRH and mental health care.

## Main narrative

### A holistic, integrated approach to healthcare delivery

Integrated care require changes to the delivery of healthcare services to promote coordination (8). It is the deliberate organization of patient care among providers to facilitate the delivery of appropriate care and information exchange (33). Integration also involves case management, where health and social care are provided at various steps along the care continuum. Integrated services may also involve the concept of a medical home, where patients and their families can coordinate care delivery with their physicians in a health facility (34). Integrated and coordinated care can also be at the (1) micro- or clinical level, targeting person-focused care; (2) meso- or organizational level, where inter-organizational services are coordinated to deliver care to a population, and/or (3) macro/system level, referring to health and social systems providing care with focus on governance, financing and policy (35).

The provision of healthcare services through integrated models is more likely to reduce cost of care, lead to better control of disease and thus improve health outcomes, reduce errors (36), improve patient-provider and provider-provider communication, as well as clarify roles and accountability (37). Integrated care depends on interprofessional collaboration to provide patient-centered care as outlined in the WHO Global Oral Health Action Plan (38). To engage in this collaboration, healthcare professionals need to be trained using updated and innovative curricula that recognize the necessity of healthcare systems to adapt to changing contexts and shifting population structure (39). Integrated care also contributes to addressing the targets of Sustainable Development Goal 3 (Good Health and Wellbeing) (40).

### Models of integrated maternal-child/adolescent health and chronic disease care

Examples of integrated healthcare models included the provision of HIV and sexual health services for female adolescents and young adults (41) and the integration of mental healthcare with physical care for life-limiting disorders such as cancer, epilepsy, diabetes and asthma (42). The use of integrated models to deliver adolescent health services may bring added benefits to ensure comprehensiveness, availability and cost-effectiveness. However, some factors need to be taken into consideration to design integrated care. These include the management of chronic disorders, the setting for service provision, the package of integrated services provided, healthcare professionals involved, the pattern of care coordination and the extent of patient involvement in care (43). Currently, there is no published model for integrating oral health with the mental, sexual and/or reproductive care of adolescents.

Robust integrated care models based on HIV healthcare systems have been reported in South Africa, Uganda, Kenya, Tanzania, Zambia, Malawi, Zimbabwe, and Eswatini (44–46). These models incorporate non-communicable disease (NCD) services into existing HIV care and have established fully integrated care systems, including at primary healthcare centers. HIV care models are highly amenable to addressing the needs of adolescents. Care models that incorporate NCD services can be scaled up to include OMSRH. However, OMSRH could also be established as a fully integrated care system. The integrated HIV care models implemented in Africa reduce stigma, which is one of the goals of the proposed OMSRH care model. An integrated one-stop shop approach to OMSRH services for adolescents could also build on existing models of integrated care that are embedded within primary maternal and childcare in African countries (47–50). Alternatively, care models that incorporate NCDs can be scaled up to include OMSRH services for adolescents. Where integration is not possible, another approach is to co-locate adolescent OMSRH services to facilitate convenient access and referrals.

### Needs assessments, approaches and procedures for integrating OMSRH services for adolescents in the dental clinic

Adolescents' visits to dental clinics can provide the opportunity to screen for additional health care needs with specific attention paid anxiety and depression (51), access to contraceptives (52, 53) and STIs (54). Behavior modification to reduce the risk of these health problems can occur through mental health counseling, comprehensive sex education, contraception counseling, and STI screening done in a supportive non-judgmental and confidential environment.

#### Oral, mental and sexual and reproductive health behavior modification

Integration models should target the modification of health behaviors that increase the risk of OMSRH problems, using a common risk factor approach (55). Modification approaches may include education and counselling on (1) oral sex, which is associated with some STIs (56, 57), (2) sex education, inclusive of education on contraceptive use, (3) oral cancer, which is linked to human papilloma virus exposure (58), (4) substance use, which may be associated with mental health disorders (59), and (5) bruxism, dentine hypersensitivity and gingivitis (60). In addition, because adolescence is a critical period marked by significant physical, emotional, and social changes (61), coupled with changes in the brain that may increase adolescents' vulnerability to mental health disorders (62), making mental health services accessible through an integrated platform, is essential. Additionally, sex education, inclusive of education on contraceptive use, is important for sexual and reproductive health.

Behavior modification in an integrated dental clinic-based OMSRH care setting draw examples from the United Kingdom's Make Every Contact Count (MECC) approach (63), where trained healthcare providers leverage patient contact to deliver healthy lifestyle messages. Studies show up to 70% increase in quit rates of tobacco smoking achieved by this method after 6 months (64). Adolescent contact with dental clinic services can likewise be leveraged to provide mental health, SRH and behavior modification services.

#### Comprehensive screening

Dentists, like other clinicians, take medical history before performing dental procedures or developing treatment plans. Integrated screening may check for the presence and/or risk factors for prevalent mental, sexual, and reproductive conditions using a set of short, validated questions. Using the Home, Education, Activities, Drugs, Sexuality, Suicide/Depression, Safety, and Strengths/Spirituality (HEADSSSS) psychosocial tool (65) when taking medical history can facilitate the early detection of mental and sexual health problems in the dental clinic. The conditions and risk factors screened for can differ between settings depending on disease prevalence in the locality, and on national and regional priorities. It is best to co-design screening tools and the overall OMSRH model with adolescents and community stakeholders to ensure feasibility, appropriateness and sustainability relevant to context.

#### Referrals

Referrals are another platform for integration of adolescent OMSRH services in Africa. For example, dentists consult with and/or refer patients to specialists to manage other NCDs. A team of pre-designated healthcare workers and specialists who co-manage adolescents with mental and sexual/reproductive health problems in collaboration with dentists, is more likely to ensure continuity of care in all three areas (66).

Oral health is an integral part of general health, and there are known strategies for incorporating adult primary oral health into general healthcare service delivery (67). Considerations for effective referrals systems for adolescents to oral healthcare are, therefore, required when designing OMSRH care, including how young clients can stay connected to health services during times of disruption. The dental clinic as the focal point of OMSRH care integration can improve accessibility and convenience of critical healthcare for adolescents, eliminating the need for separate appointments in different locations.

### Integrating OMSRH care for adolescents: benefits and opportunities

A holistic approach to the delivery of OMSRH care for adolescents in Africa should facilitate adolescents' access to (1) age-appropriate, evidence-based and culturally sensitive education on overlapping topics in OMSRH; (2) confidential, non-judgmental, adolescent-responsive oral healthcare services at dental clinics; (3) clinics that foster collaboration among healthcare professionals from different specialties, such as dentistry, psychology, sexual health, HIV care and treatment, and adolescent medicine; and (4) holistic care that is coordinated to facilitate joint assessments and shared decision-making in a rights-based approach.

The design of integrated OMSRH care models should incorporate the views and perspectives of adolescents themselves, in addition to that of caregivers, community leaders, religious institutions, and traditional healers. Employing a human-centered design approach can enhance the creation of OMSRH services tailored for adolescents and appropriate for local context. This methodology promotes the development of innovative, efficient, and person-centric healthcare solutions. Rooted in community-based participatory research, it emphasizes understanding the lived experiences of young individuals (67). The design process involves multiple cycles of engagement with stakeholders, incorporating a blend of qualitative and quantitative methods alongside specific design techniques (68, 69). This approach has demonstrated success in crafting and implementing effective sexual and reproductive health programs for young populations in Rwanda (70), Nigeria, Tanzania, Kenya, and Ethiopia (71, 72).

Lastly, advocacy is needed to influence policymakers to support the delivery of integrated OMSRH care for adolescents. This includes actualizing evidence-based guidelines by enacting health policy and allocating resources to promote and support the rights of adolescents to access comprehensive healthcare services, and to challenge discriminatory practices that limit access and uptake. Nigeria's 2024 National Oral Health Policy is one of such efforts. Nigeria's 2022 Adolescent Health Policy (73) also highlights the importance of comprehensive adolescent healthcare, and promotes resource allocation for integrated services.

### Provider roles in collaborative, integrated OMSRH care for adolescents in Africa

Integrated healthcare models have the potential to improve access to services, satisfaction, enhance health outcomes, and reduce health disparities (74). It requires a team-based approach to care management be based on comprehensive OMSRH needs assessments for adolescents using standardized tools. This requires the building of the competency of providers to develop and implement individualized care plans for adolescents screened in the dental clinics. Provider teams need training to enhance their knowledge and skills in integrated adolescent healthcare in addition to their individual fields or specialties (75). Such training should develop clinical skills and knowledge in OMSRH, and capacity to deliver youth-responsive, accessible, and culturally sensitive integrated care. Providers should also have access to continuous professional development and learning opportunities to stay updated on best practices in adolescent OMSRH care.

The training needs of adolescent OMSRH care providers can be determined using a modified version of the Well-Being Index, which evaluates the well-being of adolescents by considering various variables. This tool is valuable for pinpointing the most pertinent aspects of care for adolescent well-being in a specific context (76). The tool assesses the lifestyle habits, emotional status, social context, and mental skills of adolescents and can be modified to assess the impact of oral, sexual, and reproductive health on the wellbeing of adolescents. Data collected by this modified tool can be utilized to train healthcare providers to deliver responsive OMSRH services.

In addition, efficient referral pathways between OMSRH healthcare providers need to be established to facilitate seamless transitions and continuity of care. Standardized, evidence-based operating procedures should also be developed to enable healthcare professionals to make referrals based on the needs of the adolescent. The use of secure health information systems can facilitate referrals effectively as it promotes timely and accurate exchange of relevant information while maintaining patient confidentiality.

## Discussion

### Recommendations for the implementation of comprehensive adolescent OMSRH services in Africa

The preceding sections have raised several points to support integrated OMSRH services for adolescents in Africa. This section discusses how integrated OMSRH services support the argument for comprehensive adolescent health service delivery in general, and the importance of implementation science in the implementation of integrated adolescent-responsive services.

**Figure 2:**
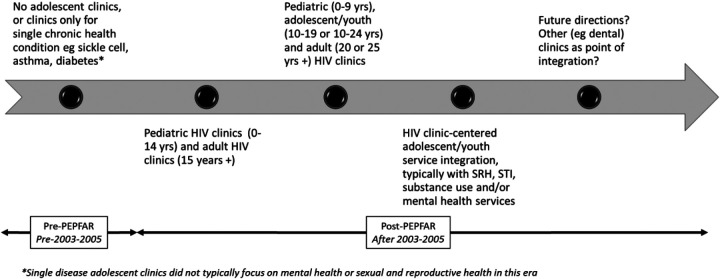
Illustration of the typical evolution of adolescent health services in a high HIV-burden African country. The establishment, funding and scale-up of structured HIV programs brought adolescent health into focus and expanded its practice in many African countries.

The acknowledgment and consideration of adolescent health in clinical practice and public health responses in many African countries were primarily driven by the HIV response. While specific clinics for the management of chronic childhood diseases such as hemoglobinopathies, diabetes, and asthma existed before HIV, the HIV epidemic elevated the prominence of comprehensive adolescent health delivery. By 2015, donor HIV funding initiatives, including the US President's Emergency Plan for AIDS Relief (PEPFAR) and the Global Fund to Fight AIDS, Tuberculosis, and Malaria, directed investments towards targeted, specialized HIV services for adolescents aged 10 to 19 and young people aged 10 to 24 (77–79). This marked a departure from the previous practice of simply disaggregating services and reporting data for pediatric (0–14 years) and adult (15 years and over) populations. Over time, with lessons learned and increased advocacy, adolescent sexual and reproductive health, tuberculosis management, and mental health, have been integrated or are being considered for integration with HIV services (6). The focus has also shifted to comprehensive adolescent health service delivery that addresses multiple health conditions through integrated services (6, 10, 75), as illustrated in Figure 2.

**Figure 3:**
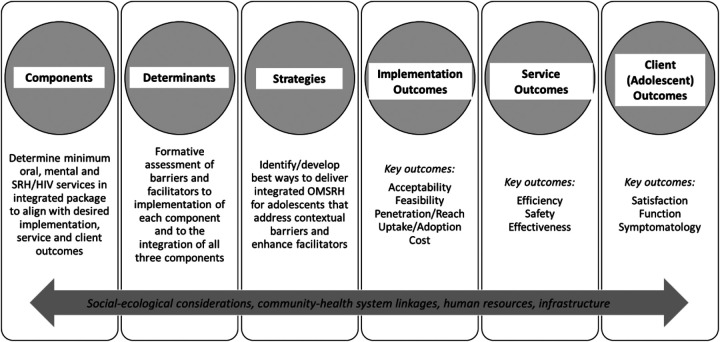
Outline of implementation blueprint for integrating oral, mental and sexual and reproductive health services in an African country. Steps include determining the minimum package of services for each component, formative/needs assessments, identifying implementation strategies to address contextual factors, and then prioritizing and measuring key implementation, service, and client outcomes.

The proposed one-stop shop approach for integrating adolescent-responsive OMSRH services in dental clinics, using Nigeria as a case study (66), requires the incorporation of implementation science methods to realize this goal. Implementation science is the study of methods and strategies that facilitate the uptake of evidence-based practices and research findings into routine practice to improve the quality and effectiveness of health services (80). It promotes health equity in the delivery of evidence-based health interventions using context-appropriate and patient-centered implementation strategies to maximize both implementation and effectiveness outcomes. Implementation science approaches for the integration of adolescent OMSRH services would require: (1) an understanding of major barriers to, and facilitators of (a) each service, and (b) the integration of all the three services; (2) the prioritization of desired implementation and effectiveness outcomes; (3) the selection of key implementation strategies to minimize barriers, enhance facilitators, and support the achievement of desired implementation outcomes; and (4) guidelines and human resources for clinical and implementation practice. This information will guide the development of an implementation blueprint as shown in Figure 3.

Some aspects of this framework reflect what has been proposed for Implementation Science-guided integrated care relevant to adolescent HIV (6), but the point of integration in our blueprint is the dental clinic, not the HIV clinic. Given the relative paucity of evidence across Africa, the proposed implementation blueprint for integrated adolescent OMSRH is largely supported by data we have generated from Nigeria. Efficacy data already exists with respect to the individual impacts of oral, SRH and mental health services for adolescents.

### Potential barriers to the implementation of adolescent OMSRH services in Africa

The integration of OMSRH services at dental clinics in African countries may face several key challenges. One challenge is the lack of policy and institutional frameworks to support the integration of oral health into general healthcare. In the short and medium term, the proposed model may have to be built on existing NCD integration frameworks to demonstrate feasibility for policy advocacy.

In addition, fragmented healthcare systems may limit cross-specialty collaboration. The proposed model leverages dental clinics as an integration hub, where structured referrals, co-located services, and shared provider training can facilitate comprehensive care. This will require allocation of already limited resources, and a shift in both healthcare provider attitudes and patient expectations. Overcoming these challenges would require concerted efforts and champions from governments, healthcare organizations, adolescents and other community stakeholders.

Furthermore, healthcare providers would need to be trained to deliver integrated adolescent-responsive OMSRH services. To address this gap, the model proposes cross-training dental health professionals to recognize and address the unique challenges faced by adolescents, including mental health concerns, sexual health risks, and reproductive health needs, all while ensuring privacy and confidentiality. In addition, they should be able to implement behaviour modification interventions, and facilitate referrals. The training would also require that healthcare providers are adolescent friendly providers, skilled in creating a supportive and non-judgmental environment, and capable of building trust with young patients. Ultimately, the goal is to empower dental professionals to serve as key players in the broader healthcare team, offering integrated care that promotes the overall well-being of adolescents.

The training of healthcare providers should take care of ethical concerns including considerations around confidentiality, consent, and cultural sensitivity. Ensuring confidentiality and privacy is paramount, as patients may be reluctant to disclose sensitive information, such as their HIV status or sexual orientation, in a setting not traditionally associated with SRH care. The implementation of robust data protection measures, including encrypted records and secure consultation spaces, to prevent breaches of patient privacy would be very important (81). In addition, clinics should adopt opt-in care models, allowing patients to make informed choices without fear of coercion or negative repercussions on their care (82). Furthermore, cultural sensitivity, and non-discriminatory services remain key concerns, particularly for sexual minority populations who may already face systemic exclusion in healthcare settings. Cultural competency training for all staff is highly essential, ensuring that patient interactions are respectful and inclusive (83). Also, psychological safety must be carefully considered, as discussing SRH topics in a dental setting may trigger distress or retraumatization for some patients (84). Healthcare providers should receive training in trauma-informed care, enabling them to recognize signs of discomfort and provide appropriate referrals to mental health professionals when needed.

Stigma and social barriers may further hinder adolescents from seeking mental and sexual health services. Embedding these services within dental clinics—a less stigmatized setting—may help normalize comprehensive adolescent care and reduce hesitation in accessing necessary support. The successful integration of NCD services into existing HIV care models offer valuable lessons in task-shifting (85), co-location of services (86), and stigma reduction strategies (87), all of which are applicable to OMSRH integration.

Access and equity are also pressing concerns, as stigma may discourage certain groups from utilizing integrated services. To bridge this gap, clinics should explore alternative service delivery models, such as mobile clinics and telehealth options, which can provide discreet and convenient care. In addition, recruiting diverse healthcare providers who reflect the communities they serve can enhance trust and engagement. In addition, the legal and ethical tensions surrounding SRH services in dental clinics cannot be ignored, especially in regions where same-sex relationships are criminalized. Providers may experience conflicts between protecting patient confidentiality and complying with restrictive legal frameworks. Advocacy for legal protections that allow healthcare workers to prioritize patient rights is essential, alongside clear clinic policies that align with international human rights standards.

Table 1 applies the Consolidated Framework for Implementation Research (CFIR) (88) in summarizing potential barriers and facilitators to implementing integrated adolescent OMSRH services in African countries. The next step is to better define the blueprint by conducting formative studies in different African settings to understand contextual determinants of the implementation of an integrated adolescent OMSRH model. A determinant implementation science framework can guide the design of this formative study. The intervention we propose (service integration) is reflected in implementation strategies such as “change service sites”, and “create new clinical teams” listed in the Expert Recommendations for Implementing Change (ERIC) compendium (89). Formative study findings will inform the design of pilot studies to better define the implementation blueprint and formulate the delivery approach for integrating OMSRH services for adolescents. The blueprint would also include details on multidisciplinary interprofessional collaboration (38), emphasizing the importance of coordinated networks and communication within the setting (90).

**Table 1:** Potential barriers and facilitators for implementation of OMSRH integration models in African countries^a^.

Potential barriers	CFIR 2.0 domain	Potential facilitators
Paucity of evidence on feasibility, implementation and effectiveness of OMSRH models	Innovation (OMSRH Models)	Implementation Science (IS) methods will provide strategies for optimizing integration and sustainability
Complexity of OMSRH integration as a novel approach will require significant adaptation of existing service structures		Increased efficiency, reduced stigma and improved service uptake for neglected areas like oral and mental health
Inconsistent data and monitoring systems limit evidence-based decision making	Inner Setting (Facilities)	Multidisciplinary collaboration that establishes referral pathways between OMSRH providers can enhance service delivery
Lack of policy and institutional frameworks	Outer Setting (Policy and Systems)	Leveraging existing NCD/HIV integration frameworks
Fragmented healthcare systems may not allow for cross specialty collaboration		Alignment with the WHO Global Oral Health Action Plan and the NCD agenda
Limited healthcare worker capacity to implement integrated and adolescent responsive care	Individuals (Implementers, Adolescents and Other Stakeholders)	Healthcare worker training to improve knowledge and capacity to implement integrated adolescent care
Adolescent hesitancy induced by privacy concerns, fear of judgement, and misinformation may reduce service utilization		Engaging champion stakeholders who have interest and influence
Stigma and social barriers are a hindrance to adolescents accessing services		
Monitoring and evaluating integrated OMRSH services require sustained effort and resources, which may be lacking.	Implementation Process	Projections and sensitivity analyses data that help to predict outcomes with different OMSRH models
		Formative research and pilot studies to test integration models before full-scale implementation
		Using an implementation science blueprint and existing standardized tools

^a^ Facilitators and barriers listed on the same row are not necessarily matched. CFIR, consolidated framework for implementation research; OMSRH, oral, mental, and sexual and reproductive health; NCD, noncommunicable disease.

Although interprofessional collaboration appears to be important for delivering OMSRH services to adolescents, the evidence regarding the effectiveness of practice-based interventions aimed at enhancing such collaboration among healthcare professionals remains limited. The existing literature is scarce, predominantly focused on high-income countries, and with no evidence on its impact for adolescent care (91). It, however, strengthens positive perception of adolescent mental health care (92), and can contribute positively to the successful outcomes of integrated OMSRH services for adolescents, especially in low middle-income countries.

## Conclusions

This article proposes an integrated model that leverages dental clinics for delivering oral, mental and sexual and reproductive health services for adolescents in African countries. There is evidence of the interconnectedness of adolescent oral, mental, and sexual, and reproductive health—and addressing these three areas through coordinated, integrated care may be a cost-effective, high impact strategy for African countries, many of which have limited resources and large youth populations. However, there remain large gaps in knowledge and implementation experience in this area. Critical research areas include primary data from needs assessments, data on different approaches to, and evaluations of integrated OMSRH care, and analyses of adolescent health outcomes in different OMSRH integration scenarios. Finally, there should be detailed information on the current structure of available oral health services in different African countries, with particular emphasis on utilization among adolescents. We recognize that dental clinics can facilitate access to integrated OMSRH care, although the degree of success will depend on evidence, much of which is yet to be generated, including the best integration models, their feasibility, appropriateness, sustainability and effectiveness. We present this paper to stakeholders for their information, consideration and critique, and to serve as a guide for those who wish to join us in the pursuit of research in this area of interest.

## Data Availability

Publicly available datasets were analyzed in this study. This data can be found here: Not applicable.
